# ISA 100.11a Networked Control System Based on Link Stability

**DOI:** 10.3390/s20185417

**Published:** 2020-09-21

**Authors:** Heitor Florencio, Adrião Dória Neto, Daniel Martins

**Affiliations:** 1 Digital Metropolis Institute, Federal University of Rio Grande do Norte, Natal, Rio Grande do Norte 59078-900, Brazil; 2 Department of Computer Engineering and Automation, Federal University of Rio Grande do Norte, Natal, Rio Grande do Norte 59078-900, Brazil; adriao@dca.ufrn.br (A.D.N.); danlartin@gmail.com (D.M.)

**Keywords:** industrial wireless sensor networks, ISA 100.11a, wireless networked control systems, link stability

## Abstract

Wireless networked control systems (WNCSs) must ensure that control systems are stable, robust and capable of minimizing the effects of disturbances. Due to the need for a stable and secure WNCS, critical wireless network variables must be taken into account in the design. As wireless networks are composed of several links, factors that indicate the performances of these links can be used to evaluate the communication system in the WNCS. This work presents a wireless network control system composed of ISA 100.11a sensors, a network manager, a controller and a wired actuator. The system controls the liquid level in the tank of the coupled tank system. In order to assess the influence of the sensor link failure on the control loop, the controller calculates the link stability and chooses an alternative link in case of instability in the current link. Preliminary tests of WNCS performance were performed to determine the minimum stability value of the link that generates an error in the control loop. Finally, the tests of the control system based on link stability obtained excellent results. Even with disturbances in the network links, the control system error remained below the threshold.

## 1. Introduction

The implementation of new communication technologies in industrial automation allows for the integration of industrial processes with greater efficiency, availability and quality. The systematic association of control and monitoring systems with communication systems generates several benefits. Wireless networked control systems acquired significant technological advancements with the rise of wireless networks, advanced control, embedded computing and cloud computing.

The connection of devices spatially distributed for different purposes with wireless networks provided a great increase in applications using wireless sensor networks (WSNs). However, critical process control constraints defined some limits of WSN technology. Industrial wireless sensor networks (IWSNs) are a specific field of WSNs, which takes into account reliability constraints, timing deadlines and critical nature of industrial applications.

The industrial wireless sensor networks are being used in various branches of industry: area monitoring, structural monitoring, disaster prevention and control systems. Many applications in different monitoring and control systems are said to be safety related. In such kinds of systems, a system failure may put people in danger, lead to environmental damages or result in economic losses [1]. The guarantee on packet reception and reliability must also be provided for feedback control systems to operate properly. There is also a need for extensive measures to be implemented to counter the uncertainties of wireless means of communication [2]. System performance evaluation parameters are required to ensure these extensive measures.

The development of wireless networked control systems (WNCSs) is fundamental in Industry 4.0. The development of both intelligent manufacturing equipment and intelligent control systems is a priority in the machine tools sector. Additionally, the priorities in the area of IT include the Internet of Things (IoT) and its applications, including industrial control [3]. Since the cyber-physical systems represent the integration of physical systems with computing and networking capabilities, WNCSs are an important class of cyber-physical systems in Industry 4.0, in which physical processes are controlled using wireless sensors, actuators and controllers [4].

A WNCS connects sensors and actuators of a plant to a controller via a wireless network, which has several critical communication channels. These links are classified as critical because they are part of several closed-loop control systems. Thus, the system design must also take into account an evaluation parameter for these links. Link stability is an appropriate factor for analyzing links that carry information from sensors to the controller.

In this paper, we present an ISA 100.11a wireless network control system. The control system receives two level measurement values, but the choice of the level value depends on the stability of these two links. Link stability assesses the performance of the link from samples of the received signal strength. A permanent monitoring of the link stability guarantees the regularity of the control system. The networked control system is implemented with ISA 100.11a wireless sensors, network manager, gateway, controller and wired actuactor (pump).

The remainder of this paper is organized as follows: Section 2 discusses some related works about WNCS and link stability on wireless networks. In Section 3, the overall the system architecture is described. Section 4 describes the software implemented in the controller of the system. All system tests and results are presented in Section 5. Finally, conclusions are stated in Section 6.

## 2. Related Works

### 2.1. Wireless Networked Control Systems

The industrial networks are part of the structure of automation systems. They allow the communication of all instruments in the system, including sensors, actuators, controllers and data acquisition stations. The wireless sensor networks have been used in many monitoring applications for various physical phenomena, such as temperature, flow, level, vibration, humidity and pump analysis [5]. With the advent of the Industrial Internet of Things (IIoT), the availability of fast, secure and reliable communication networks deployed within factories and connecting all the elements of industrial control systems became a requirement [6]. However, deployment of wireless communication in the control systems creates new obstacles to overcome, such as the need of designing the control parameters associated with the network parameters.

Wireless networked control systems allow all or some of the measurement and control signals to be transmitted over wireless channels. There are approaches in which both signals the one from sensors and the one sent to the actuators travel through wireless communication technology. However, there are other approaches that use wireless and wired signals. The data that flow between sensor nodes and controllers are not necessarily symmetric in WNCSs [7,8,9].

The objectives of the networked control system are to ensure that the closed-loop system has desirable dynamic and steady state response characteristics, and that it is able to efficiently attenuate disturbances and handle network delays and loss [7]. The main communication problems are the delay and the packet loss rate, which directly influence the reliability of the system. The delay problem may greatly reduce the performance of the control system, so that the stability of the system is narrow.

There are several industrial wireless communication protocols that allow different configurations of parameters and structures. Likewise, there are several control loop techniques with several variations. A proper model must consider the parameters of both control and communication. Efficient integration of communication and control has been identified as a high-impact challenge for the next generation of industrial automation systems [10]. A more integrated approach is necessary in order to design systems that systematically model parameters between the communication and control systems.

Determining the optimal parameters for minimum network cost while achieving feasibility is not trivial because of the complex interdependence of the control and communication systems. WNCSs require novel design mechanisms to address the interaction between control and wireless systems for maximum overall system performance and efficiency [7]. Several researches have being developed to model, evaluate and validate wireless network control systems [11].

Park et al. [7] presented and explain many criticial system variables. There are critical variables both in the control system and in the wireless communication system, which are closely linked. For instance, the control system defines the sampling period and the communication protocols determine the retransmission mechanism in case of failure. Therefore, the maximum retransmission period (communication system) must be determined together with sampling period (control system). The critical variables in the communication aspect are packet delay rate and packet loss rate. Additionally, in the control system aspect, the variables are sampling period, message delay and message dropout [7].

The delay time is a parameter used by several researchers to model WNCSs with different approaches [5,10,12]. Shi et al. [5] modeled the network time delay in the multi hop network caused by the S-MAC communication protocol. The model also considers the controller model in a WNCS. Araujo et al. [10] took into account the delay in the sensors’ and actuators’ links and modeled a solution to compensate for the delay. The paper categorizes two types of delays: delays in the access to the communication channel and delays due to the transmissions and computation at the controller. In [12], the model is based on research area of delay-constrained wireless communication. This paper analyzed a WNCS with multiple control systems sharing a commom wireless channel.

Many researchers have implemented WNCSs with simulators only, which causes the absence of experimental tests with instruments of the manufacturers in the market. By way of example, Horvath et al. [4] presented a simulation framework which includes a realistic model of the physical layer with multi-channel frequency-hopping mesh networks. The simulation framework is evaluated by implementing a WNCS based on WirelessHART.

Park et al. [7] and Araujo et al. [10] presented experimental tests with wireless instruments in the level control system of coupled tanks. Both instruments used in the tests are Telos nodes [13]. Ahlén et al. [14] presented an implementation on an industrial process at the Iggesund paper mill. All control loops were implanted using wireless sensors and actuators. Additionally, the ABB AC800M controller received and sent information to the instruments by communicating with the network gateway via Profinet. The results indicate that it is feasible to use wireless control for continuous production. Ahlén et al. [14] ensured that it is possible to reach the desired availability with wireless instrumentation compared with the wired instrumentation.

In this study, we implemented a wireless networked control system based on ISA 100.11a instruments from the manufacturer Yokogawa Electric. In addition to the delay time, the packet loss rate in communication links is also an essential parameter in the evaluation of the communication system. As such, this study evaluated the implementation of a WNCS based on the evaluation of the links that are part of the control loop. The stability of the links is the factor used in the system proposed in this work.

### 2.2. Link Stability in IWSN

A wireless sensor network contains several instruments located in different locations, which generate different communication links. Communication between two devices can have more than one link. However, each link has its characteristics and can be affected differently by interference. One way to evaluate these links is by the link stability factor. The link stability should not be confused with the stability of the control system, the concepts are significantly different from one another.

Some researchers claim that link stability indicates how stable the link is and how long it can support communications between two nodes [15]. For others, the link stability means the link will sustain for a long time and does not break regularly [16]. Overall the link stability indicates the level of variation of the link with respect to the level of noise and the rate of packets lost.

In paper [17], we presented a study about link stability in IWSN. Link stability is defined by the variation of received signal strength (RSS) and packet delivery rate (PDR). An unstable link presents a high variation of signal attenuation and low packet delivery rate. A stable link has no signal attenuation variation and has a high packet delivery rate [17]. Table 1 is formulated to present the selected papers in [17] and their parameters used to define stability.

Many papers use the distance between nodes and expiration time because they are applied to mobile networks, which have a high level of mobility. Models that use link expiration time do not consider the instability during the period that the links are active on the network. The appearance of several interferences is common, even with the device in the same position in IWSN.

In industrial wireless sensor networks, the devices are typically distributed at fixed locations without mobility, and thus the links remain active throughout the period of operation. Therefore, the model of this work does not consider the link expiration time. Additionally, the distance between nodes parameter is used indirectly in the acquisition of the received signal strength parameter.

The link stability function is based on the variation of the received signal strength. It was necessary to create a factor that indicates the degradation of the signal to calculate this variation. This factor is generated from previous samples of the received signal strength and the current value. The entire process of generating link stability is detailed in Section 4.1. A link with low stability directly implies the performance of the control system.

In this study, the link stability was be used in the controller program to choose the measurement value used in the control technique. However, the evaluation of the implementation of the network control system is the main objective of this work. The next sections detail the system implemented.

## 3. System Architecture Design

The system architecture consists of a controller to receive data from the sensors and send the signal to the actuator. Data are collected through the ISA 100.11a network gateway, which interfaces with all measurement elements. However, the only actuation element in the system uses a wired signal. The systems in papers [4,32] also use the wired signal from the actuator.

The system implements a tank level control loop with wireless instrumentation. The system configuration allows the controller to choose the process variable (PV) value between the LD01 sensor route and the LD02 sensor route. Thus, the stability metric of these links is the parameter that defines the choice of PV. Next the controller runs the program and sends the wired signal to the pump.

**Figure 1:**
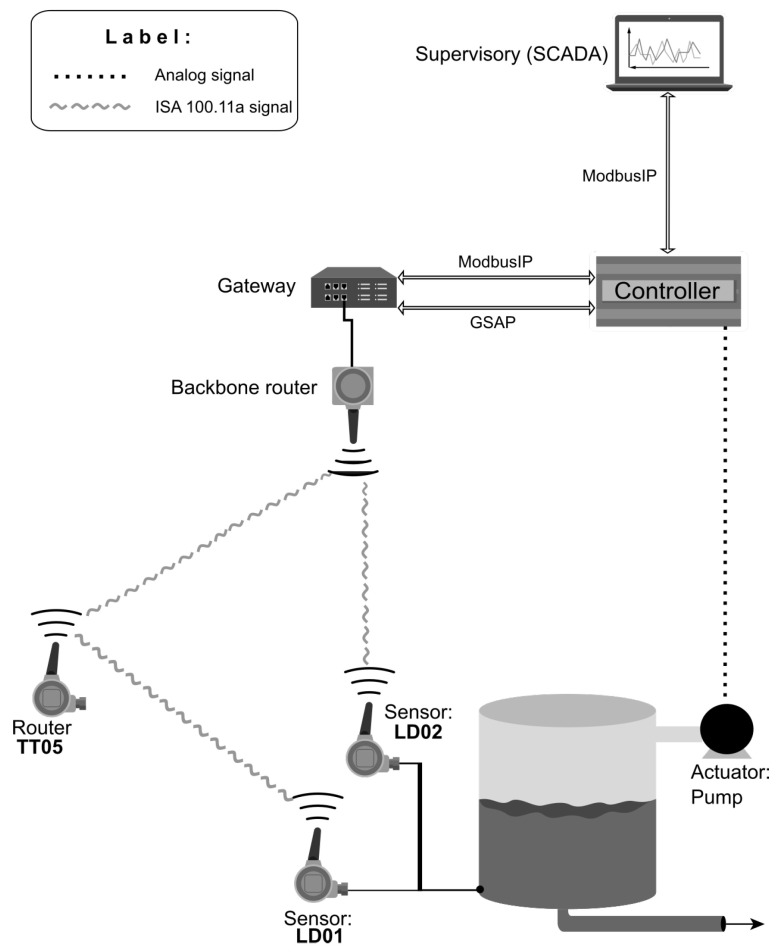
ISA 100.11a network control system architecture.

The controller provides two communication interfaces with the ISA 100.11a gateway to collect data from the links and measurement variables of the instruments. In addition, an interface with a supervisory system is provided to enable monitoring of variables. Figure 1 shows the system architecture.

It is possible to notice in Figure 1 that there are two differential pressure sensors to measure the tank level (process variable): LD01 and LD02. These sensors measure the same differential pressure value. That means the controller can receive the same process value from both routes to control the tank level.

### 3.1. Level Control System

The control system is composed of two tanks coupled, a water basin, two level sensors and a pump. The liquid in the lower tank flows to the water basin and a pump is responsible for pumping water from the basin to the upper tank. The liquid in the upper tank flows to the lower tank. There is a sensor in a each tank for measuring the level of tank.

The system in this project used only an upper tank. The tank shown in Figure 1 represents the upper tank of the double-tank coupled. However, the two sensors LD01 and LD02 were placed at the same measuring point. The integration of these measurements from the tank with the controller was performed through the ISA 100.11a wireless network.

### 3.2. ISA 100.11a Network

Nowadays, industrial wireless networks are part of the structure of automation systems. They allow the communication of all field instruments, including sensors and some actuators, with controllers and data acquisition stations. Some protocols define the rules and techniques for wireless communication at the sensors, actuadors and controller, such as IEC 62734 (ISA 100.11a) [33].

The ISA 100.11a architectures contain elements that perform information transmission, information routing and network management at different levels. Each network is formed with nodes and composed of a processing unit, a radio, memory, a data acquisition board and a battery. Currently, regardless of topology, all networks need a central element to concentrate information from all nodes: the network manager. The manager receives and sends the data packets to the nodes, and manages all links formed in the network. Within end-to-end communication, at least one of the elements is the manager, either by receiving the packet from a measuring element or by sending the packet to an actuation element.

**Figure 2:**
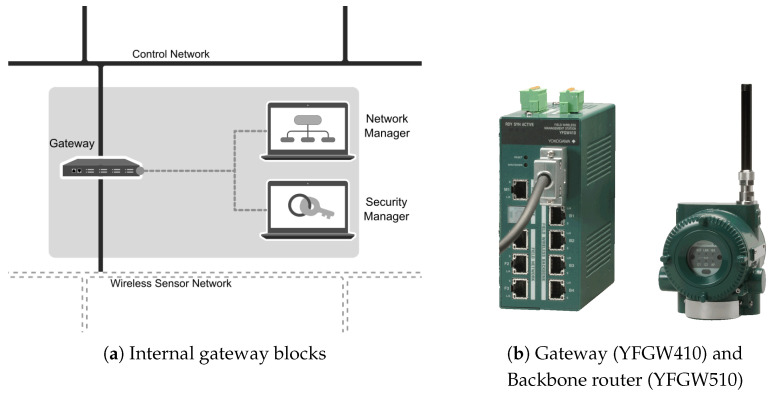
Gateway, network manager, security manager and backbone router.

The network manager is not responsible for the junction authorization of a new device to the network. The tasks of managing security keys, such as authenticating, generating and storing, are the responsibilities of the security manager. Physically, the two managers are integral logical parts of the gateway element, as shown in Figure 2a. In this work, the network manager, security manager and gateway were implemented in a single management station: YFGW410 (Yokogawa Electric) [34]. The first device in Figure 2b is gateway YFGW410.

The second device in Figure 2b is the backbone router YFGW510 (Yokogawa Electric) [35]. The wireless devices communicate with the gateway and therefore with the managers through backbone router, which operates as an access point of wireless network.

The field devices on the ISA 100.11a network can be routers or non-routers. The use of routers in networks can transmit their data and their neighbors’ data to the manager, thereby increasing the redundancy and availability of the network due to alternative paths generated.

**Figure 3:**
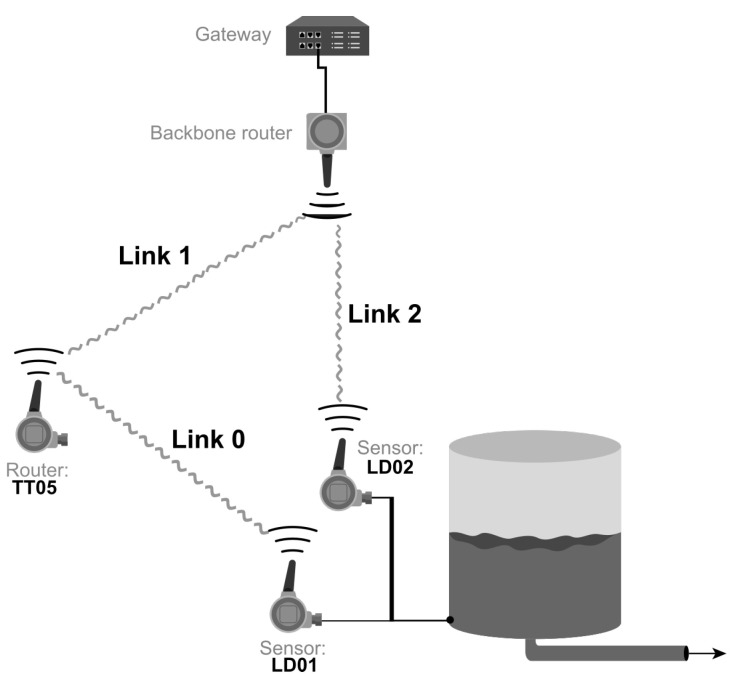
The system links.

The ISA 100.11a network of the Figure 1 architecture includes only three instruments: two differential pressure sensors (LD01 and LD02) and a temperature transmitter, which operates in the router mode of the LD01 instrument. Unlike the LD01 sensor, the LD02 instrument has a direct link to the gateway. Figure 3 shows the number and nomenclature of the system links.

The links created in the system configuration are:

- textbfLink0: LD01 → TT05.
- textbfLink1: TT05 → Gateway.
- textbfLink2: LD02 → Gateway.

The LD01 sensor route is composed of link0 and link1, and the LD02 sensor route is composed only of link2. The purpose of insertion of the router TT05 in this work was to be able to cause a forced attenuation in the antenna of this instrument and then analyze the influence on the level control loop. Therefore, it is mandatory that the controller be able to collect the link stability information before executing the control logic and sending the signal to the pump.

### 3.3. Controller Board

The controller must be able to collect the measurement values of the sensors (LD01 and LD02), calculate the link stability levels, execute the level control program, control the pump and send the monitoring data to the supervision system.

The link stability metric is generated from the RSSI data of the links. Hence, the controller will collect these data from the links through a communication interface with the gateway. The controller must send requests to the gateway following the GSAP (gateway service access point) specification. The system collects the measurement values from the sensors through a Modbus TCP communication interface with the gateway. Finally, the sending of data to the supervision system also uses a Modbus TCP interface.

**Figure 4:**
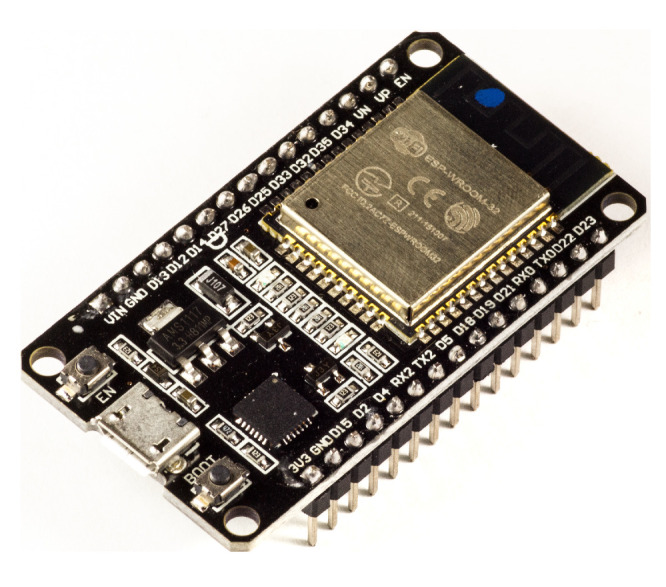
ESP32 controller.

Due to the high communication and processing capacity and the need for a WiFi module to implement Modbus TCP and GSAP commands, the system controller chosen was the ESP32 microcontroller from the company Espressif. Figure 4 shows the ESP32 controller.

ESP32 is a single chip designed with ultra low power technology that incorporates microprocessing, memory, peripherals and communication modules (WiFi and Bluetooth). The main features that distinguish it from other platforms used in embedded systems are: two processing cores, a 160 MHz clock, an integrated Bluetooth module, a flash memory expandable to 32 GB, 36 GPIO pins and 18 channels of analog-to-digital converters [36].

This controller has been used in several IoT applications due to its processing power and low power consumption. It is possible to define a completely wireless solution using the ESP32 module, which integrates the IEEE 802.11 network protocol with the IoT architecture [37,38].

**Figure 5:**
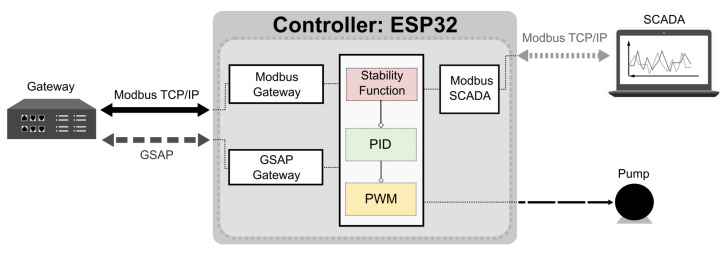
Controller modules.

Two communication modules between controller and gateway were implemented: Modbus TCP communication and GSAP communication. The Modbus TCP interface is responsible for acquiring the process variable data from the LD01 and LD02 sensors. The GSAP module requests the information from the links, focusing on the RSSI values used in the link stability function. Figure 5 shows the modules implemented in the ESP32 controller.

The link stability module implements the link stability analytical model and defines which PV value will be used in the control logic. Finally, the pump receives a pulse-width modulation (PWM) signal resulting from the program that controls the tank level.

#### 3.3.1. Modbus TCP Communication

The Modbus protocol is an industrial communication protocol at the application layer that follows a master–slave topology in order to perform the communication between devices. Only one device, the master, can initiate request–response messages to other devices (slaves) by sending a query to an individual slave or sending a broadcast query to all slaves. In the case of Modbus TCP/IP, the slave address is identified by an IP address [39].

It is then possible to use Modbus over serial protocols (e.g., RS-485) or TCP/IP protocols on Ethernet. In any case, the message structure is always the same. The Internet community can access Modbus at a reserved system port 502 on the TCP/IP stack. Modbus TCP/IP is mostly used in the data sharing between the field device level (e.g., PLC, CAN J1939 to the Modbus Gateway) and the SCADA system level. Modbus TCP/IP as a protocol could support communication between field devices via TCP, i.e., between sensors, actuators and PLCs [39,40].

There are two Modbus TCP communication modules in the controller: communication with the ISA 100.11a gateway and communication with the supervision system (ScadaBR).

In the Modbus TCP communication module with the gateway, the communication master is the ESP32 controller, which makes the requests, and the communication slave is the gateway. The slave’s Modbus memory mapping (gateway) contains the PV values of the two sensors, as shown in Table 2.

The controller also provides a Modbus communication with the ScadaBR supervisory application to supervise the control system [41]. Only the main variables of the control loop and the network are monitored.

Unlike the other Modbus module, the ESP controller is the communication slave in the interface with the ScadaBR application (the communication master). The mapping of the ESP32 Modbus memory, as shown in Table 3, only contains variables of the type holding registers. Some variables occupy more than one memory position (offset) due to the representation as a float data.

ModePID, PV1, SP, MV and PV2 are variables of the control loop and the other variables represent the behavior of the network links. All of these variables that describe the network’s performance were collected from the gateway through GSAP communication.

#### 3.3.2. GSAP Communication

The ISA 100.11a standard describes an access point interface to gateway services: GSAP (gateway service access point). This service is generic and should be used as a common interface above the application layer of the protocol [33].

GSAP is a specification that defines the support features that allow a communication interface between the ISA 100.11a network and an external network. The standard describes how to implement messages from the GSAP specification using the 15 objects and services described in the standard. There is no complete detail, but a reference to help understand the commands [33].

The commands are implemented using objects from the protocol application layer. Each service accesses a specific type of network manager object. However, there are several commands that can manipulate these objects. Table 4 presents some GSAP services described in the ISA 100.11a standard.

In this work, only the commands G_Session_request and G_Neighbor_Health_Report request were implemented. The first service (Session) performs the opening of the GSAP session with the gateway. The second service (Neighbor_Health Report) is responsible for requesting data from neighbors on a field device on the network.

Each service has specific fields in the request and confirmation messages. The programmer must understand all fields in the package to be able to communicate with a gateway. In addition, it is only possible to request a *Neighbor_Health Report* service if the session is already open.

In order to exemplify a GSAP service request, Table 5 presents the fields of the command *Neighbor_Health_request* with the respective example values.

The command *Neighbor_Health_request* returns an object of type *NeighborHealthReport*, which stores all values about the neighbors of a given instrument on the network. The object returns a vector of elements of type *NeighborHealth*: *neighborHealthList[]*. The *NeighborHealth* structure stores all the data from the link between the transmitter, identified by the Network address field in Table 5, and the neighbor (receiver), identified by the *networkAddress* of the *NeighborHealth* structure.

The GSAP driver sends the opening session command and the command *Neighbor_Health request* for each link in the system Figure 1, thereby obtaining all data from the network links.

## 4. Software Implementation

The software includes all the modules shown in Figure 5. The stability function, and PID and PWMmodules are part of the main logic of the controller. The implementation of the link stability metric generation uses the values of the received signal strength indicator (RSSI). The stability values of the links are used in the selection of PV. Finally, the controller performs the PID control technique.

### 4.1. Method of Link Stability

The method provides an evaluation of the link stability of a IWSN. As mentioned in Section 2.2, in the context of an environment susceptible to different types of interference, the link stability is essential to evaluate network performance.

The lower the stability of the link, the higher the variation of the attenuation in the reception signal, and consequently, the greater the instability in the delivery of packets [17].

The method is based on a linear function proportional to the variation of the received signal strength (RSS) and the packet delivery rate (PDR) within a set of samples. This metric is generated from previous samples of RSS and current value of RSS and PDR. Equation (1) presents the link stability factor.

(1) $$LinkStability=PDR*(1-0.04^{MME_{RSSRatio}})$$

The variable $MME_{RSSRatio}$ is the exponential moving average of the standard deviation values of the attenuation ratio (*RSSRatio*). The purpose of using the moving average is to generate a filter to reduce the influence of outliers, and consequently, present a factor with greater smoothness.

The moving average is based on a set of samples of the variable ($\sigma_{RSSRatio}$); it has a size that varies when a new sample appears. The amount of samples in a data window can be defined by the user when implementing the method.

The first variable to generate the Equation (1) is the rate of attenuation of the received signal: *RSSRatio*. This variable relates the current RSS value to the previous values. The purpose is to relate the current strength value to the signal attenuation in the last samples. The Equation (2) shows the *RSSRatio*.

(2) $$RSSRatio=\frac{RSS_{i}}{RSS_{max}}$$

The variable $RSS_{i}$ represents the current value of RSS and the $RSS_{max}$ represents the maximum value of the RSS sample set. The greater the variation of *RSSRatio*, the greater the instability of the received signal strength. Thus, the value used to calculate the moving average is the standard deviation of the RSSRatio values.

**Figure 6:**
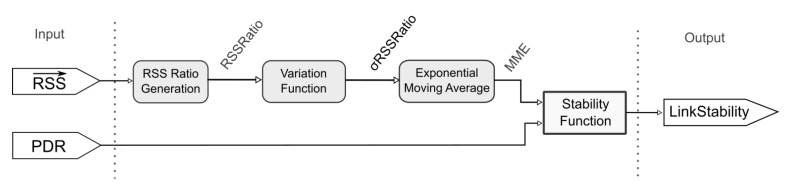
Overview of the method for link stability evaluation.

As shown in Equation (1), the exponential function with fixed base and exponent MME is used to smooth the factor that multiplies the PDR. This smoothing avoids the generation of very low values of stability when the MME variable becomes very low. Figure 6 shows the flowchart for the entire method.

Part of this method was detailed and published by Florencio and Neto [17]. The link stability metric is able to detect instabilities in the links, which can cause an increase in the packet loss rate, and consequently, considering a networked control system, take the system to an unstable region [17].

### 4.2. Control Program

The control technique is an proportional–integral control (PI), which operates at a sampling rate of 100 ms and with gains of KP = 1.6 and KI = 0.15. As the controller modeling and tuning processes are not part of this work, standard values of other works developed with the same tank system were used.

Link0, shown in Figure 3, is the primary link to acquire the level value (PV1). However, if the stability level of link0 is equal to or below a threshold, the controller must select the PV value of the alternative route: link2 (PV2).

The control program must perform the steps listed below.

1. Read the value of PV1 (module: Modbus TCP communication with Gateway);
2. Read the value of PV2 (module: Modbus TCP communication with Gateway);
3. Read the SP value (module: Modbus TCP communication with SCADABr);
4. Read link data (module: GSAP communication with the gateway);
5. Calculate the stability value of link0 (module: stability function);
6. Calculate the stability value of link2 (module: stability function);
7. Select the PV value from the stability values (LinkStability0 and LinkStability2);
8. Execute PI control (module: PI Control);
9. Send the MV value (PWM signal) to the pump;
10. Update data for the supervisory system (module: Modbus TCP with ScadaBR).

The pseudocode Algorithm 1 presents an overview of the lines of code implemented in the ESP32 controller for level control based on the link stability metric.
**Algorithm 1:** Algorithm of the controller program.1:PV1 = modbusGW_request(1, 13, 2);2:PV1 = (double) PV1;3:PV2 = modbusGW_request(1, 34, 2);4:PV2 = (double) PV2;5:SP = (int) modbus_scada.Hreg(HREG_SP);6:gsap_requestLinks();7:stability0 = func_stability0();8:stability2 = func_stability2();9:**if** stability0 > thresholdStab **then**10:    PV = PV1;11:**else**12:    PV = PV2;13:**end if**14:MV = pidTank.Compute(SP, PV);15:PWMvalue = (int) MV;16:analogWrite(PUMP, PWMvalue);17:update_scadabr();

Each line or group of lines of code performs a step from the control program. The modbusGW_request(x, y, z) commands in lines 1 and 3 request the PV variables for each sensor, where x is the slave ID (master–slave communication), y is the address of the variable in the slave’s Modbus memory mapping and z is the size of the variable (multiples of 16 bits). Hence, as the communication slave is the gateway, line 1 requests the PV value, mapped in position 13, with a size of 32 bits.

Unlike the communication of the ESP32 controller with the gateway, the controller is the slave in the communication with the between ScadaBR and controller. The modbus_scada.Hreg(x) command reads the value contained in the x position of ESP32 Modbus memory. This memory location is written in the supervision application (ScadaBR), or rather, by the operator in the Modbus writing commands.

Line of code 6 collects the network link data used to generate the link stability factor. This command updates the following code variables: RSSI0, RSSI1, RSSI2, DPDUTx0, DPDUTxFail0, DPDUTx1, DPDUTxFail1, DPDUTx2 and DPDUTxFail2. Some of these variables are used by the stability function in lines 7 and 8.

After generating the stability values, code lines 9 to 13 ensure that the LD01 (PV1) sensor value will only be used by the PI control if the stability is greater than the minimum stability threshold. Otherwise, the value of the LD02 sensor will be used by the PI control.

Finally, ESP32 performs PI control on code line 14 and sends the signal to the pump (code line 16). At the end, all data from the control loop and the network are updated in Modbus memory in order to transfer to the ScadaBR supervisory.

## 5. System Performance Evaluation

### 5.1. Implementation

A system has been developed to evaluate the performance of the wireless networked control system with ISA 100.11a devices. All elements of the architecture (Figure 1) are present in the system, as described below.

**Figure 7:**
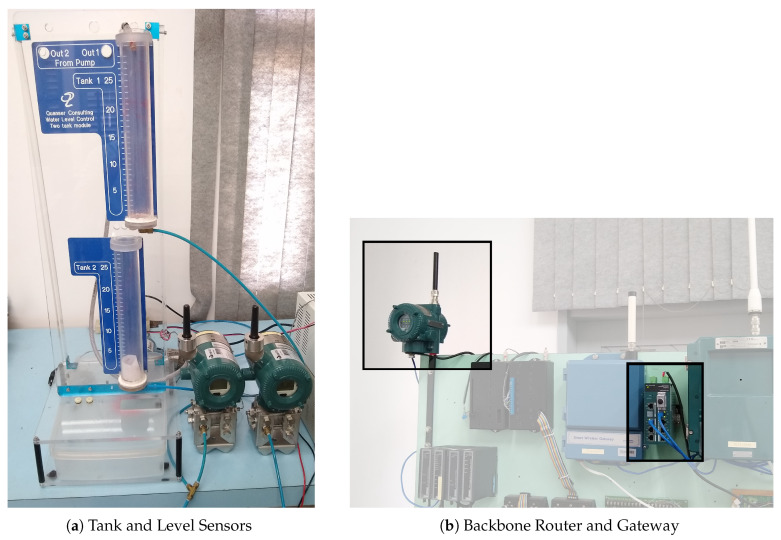
System in the industrial network laboratory.

- Controller: ESP32;
- Gateway: YFGW410—Yokogawa Electric Corporation (Figure 2b);
- Access point or backbone router: YFGW510—Yokogawa Electric Corporation (Figure 2b);
- Router TT-05: YTA510 (temperature transmitter)—Yokogawa Electric Corporation;
- Level Sensor: EJX110B (differential pressure transmitter)—Yokogawa Electric Corporation;
- Actuator: Water Pump 12V;

The tank and level sensors were placed on a test bench in the Industrial Network Laboratory, as shown in Figure 7a. The backbone router and the gateway were located at a distance of 3 m from the level sensors within the same laboratory. The TT-05 router is the only instrument that was distant, in the external area, in order to perform the signal attenuation tests.

The instruments highlighted in Figure 7b are the backbone router and the gateway.

### 5.2. Wireless Network Level Control System: Preliminary Tests

Tests of the level control system without considering the link stability metric were performed in order to analyze the influence of stability on the control error with the system in steady state.

The methodology used in these first tests is described below.

1. Start ISA 100.11a network gateway, backbone router and instruments.
2. Launch the SCADA application.
3. Start the controller.
4. Determine the setpoint at 100 mm.
5. Execute the PI control with the PV value of the LD01 sensor.
6. Wait for the control to reach the steady state with 1% error.
7. Cause a change in attenuation in the signal of link0.
8. Collect data and analyze the influence of link failure on the controller.

The main objective of these tests is to cause a variation in the attenuation of the transmission signal of the LD01 sensor to verify its influence on the steady state of the control loop.

Step 7 of the methodology is performed only after the system reaches a steady state, considering a error of 1%. Thus, it is possible to infer that the errors that arise in the control loop are due to failures in the network link.

Seven tests were carried out with a minimum duration of 20 min, considering the time to start the controller, determine the set point value and wait for the system to reach the Steady State.

In order to present a better overview of the data in this paper, data from three tests are presented: test 01, test 02 and test 03.

#### 5.2.1. Test 01

**Figure 8:**
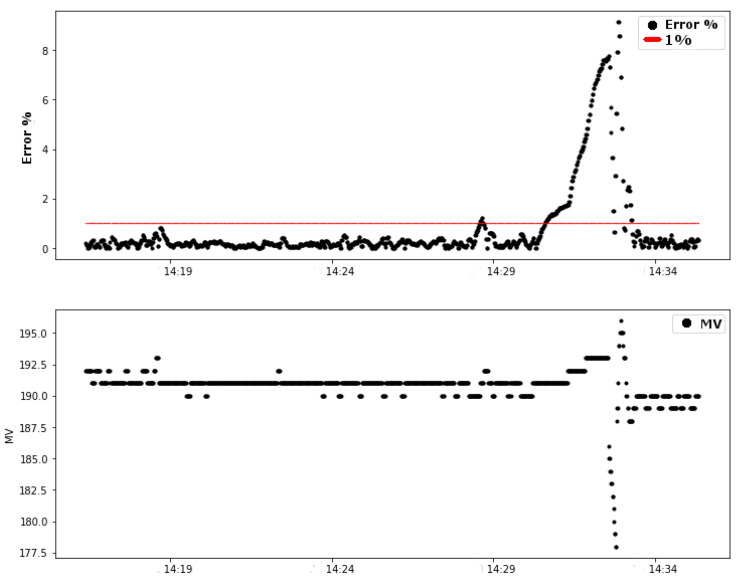
Test 01: Variables of the control loop (error and MV).

The first graph of test 01 shows the variables of the control loop: error (difference between the process variable and the setpoint value) and MV (manipulated variable). The controller calculated the value of the output of the PI control (variable MV), in a range from 0 to 255, to send a pulse width modulation (PWM) signal to the system actuator: the pump. Figure 8 shows the data for these variables during the test.

It is possible to observe that approximately in the time period between 14 h 30 min and 14 h 33 min the absolute error in the steady state of the control system exceeded the limits of 1%, reaching a value close to 9% of error. This change in the error naturally caused a change in the pump signal (MV), shown in the second graph of Figure 8. The red line indicates the limit of 1% error.

**Figure 9:**
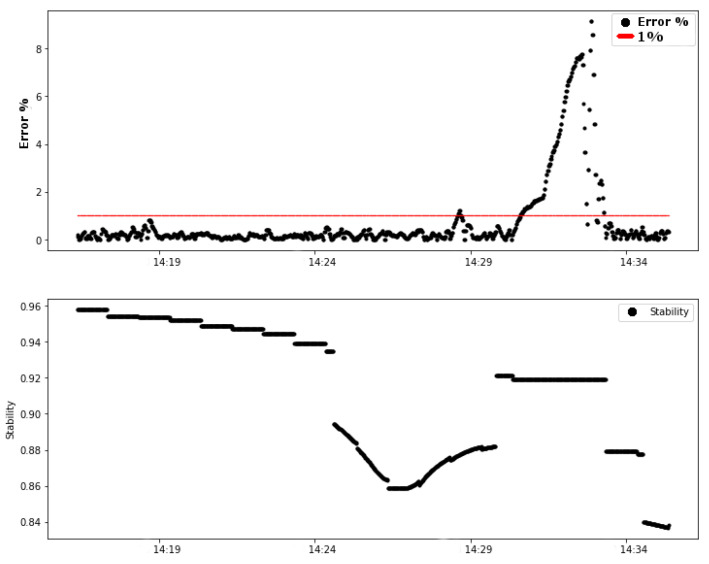
Test 01: Error (%) and Link0 stability.

An attenuation on the link is forced by changing position and inserting structures that degrade the signal transmitted on the link. Figure 9 shows the relation between the link stability value and the controller error. The analysis of the influence of stability on error is discussed in this section.

It can be observed from Figure 9 that the variation of the link stability occurred before the variation of the control error. The vertical red dashed line indicates the moment when the forced attenuation of the link started. From these graphs, the previous influence of the link stability factor on the system error was analyzed.

#### 5.2.2. Test 02

**Figure 10:**
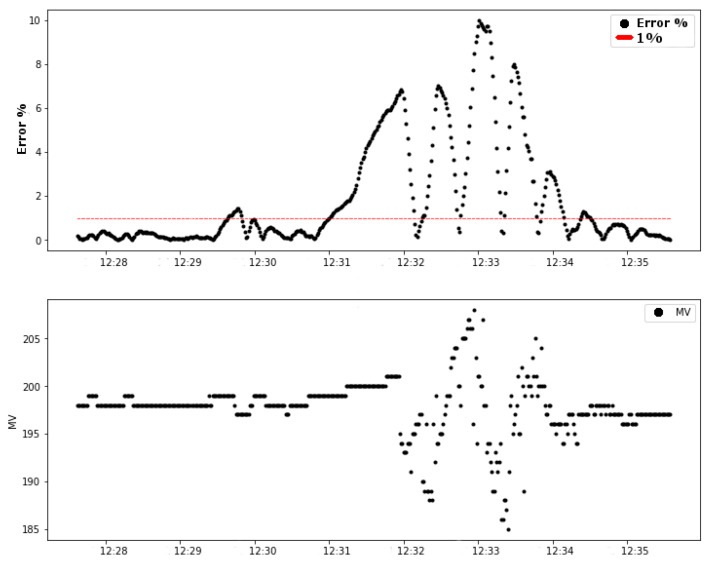
Test 02: variables of the control loop (error and MV).

Test 02 also shows a change in the controller after the link attenuation. It is important to remember that the control was already in the steady state. The values of the absolute error also exceeded the limit of 1% defined as a system requirement, as shown in Figure 10.

**Figure 11:**
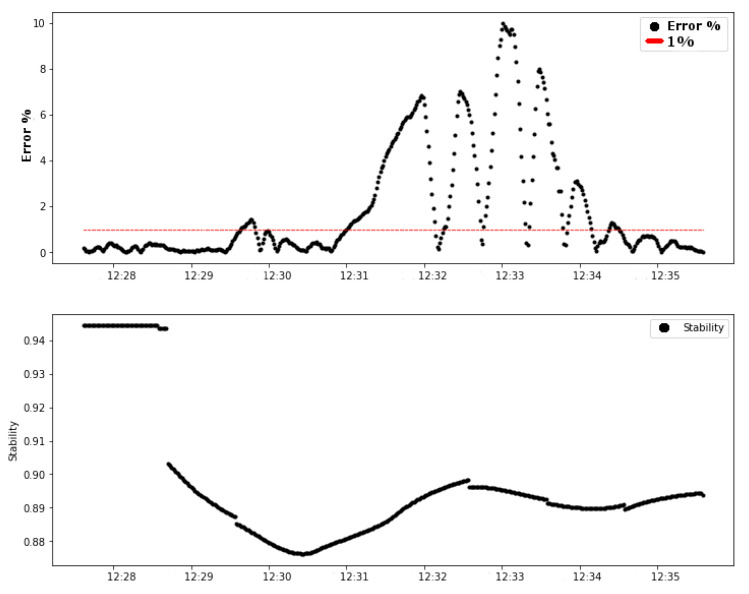
Test 02: error (%) and Link0 stability.

The same behavior of test 01 happened in the second test. There is a variation in the link stability, shown in the first graph in Figure 10. Additionally, then, the control error increased in the period of time following reducing the link stability value. These behaviors of these variables are presented in the graphics of Figure 11. The dashed vertical lines in the graphs indicate the moment of the beginning of the forced attenuation.

**Figure 12:**
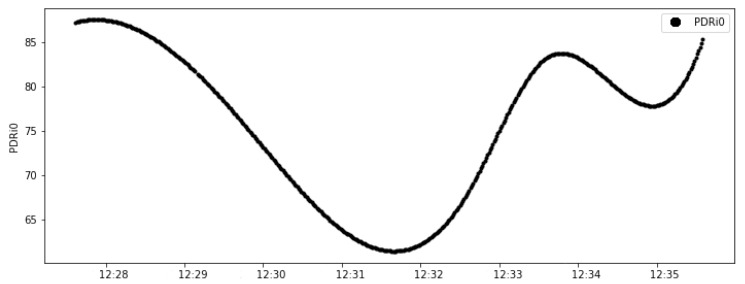
Test 02: Link0 PDRi.

An essential step in this analysis is the verification of the rate of instantly packets delivered ratio (PDRi). The relation between the PDRi of the link and the control error is shown in the graphics of Figure 12. The variation of the control error occurred later with the increase in drops packets.

The data presented in Figure 11 and Figure 12 prove that the reduction of the link stability caused a variation in the packets delivered ratio, which, consequently, increased the absolute error of the control system.

#### 5.2.3. Test 03

**Figure 13:**
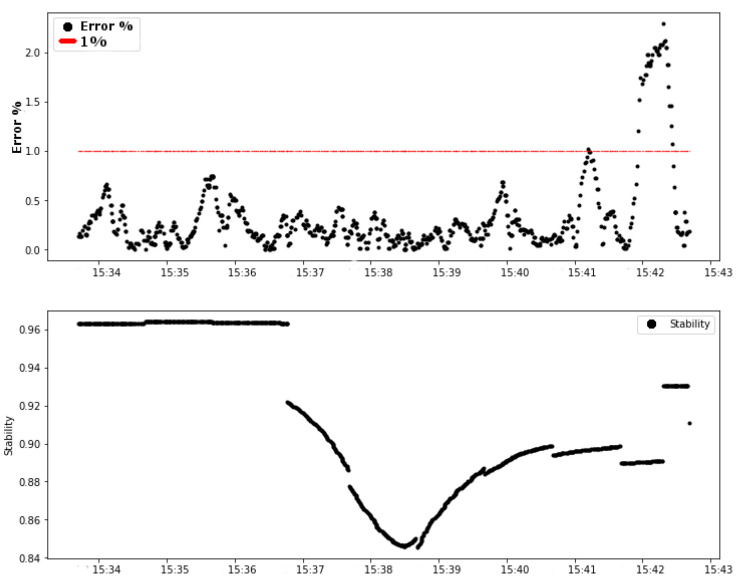
Test 03: error (%) and Link0 stability.

The results obtained in test 03 support the same conclusion as the other tests, as shown in Figure 13. The link stability factor provides a prognosis or even a prediction of the behavior of the control system in the next few minutes.

#### 5.2.4. Preliminary Test Results

From an examination of the preliminary tests it becomes apparent that the link stability factor allows a prediction of the change in the control system error.

The second graphic in Figure 13 (test 03) shows that, in approximately 15 h 36 min 40 s, the link stability decreased to around 0.92 (92%) and remained decreasing until about 0.85 (85%). In approximately 15 h 41 min 40 s, 5 min after the decrease of the stability level, the error started its ascension until it got close to 3% error.

The values of reducing the stability factor and the time between the stability variation and the increase in error are essential to use the link stability as a decisive factor in the control loop. Thus, a summary of the data from the tests performed is presented in Table 6.

The third column of the Table 6 contains the values of the median of the reduction curve of the link stability factor. Thus, the median of these values was calculated. The median of the stability values during the reduction is 89%.

### 5.3. Wireless Network Level Control System Based on Link Stability

In the preliminary tests performed, the controller received the PV value from the LD01 sensor and executed the control logic. However, failure periods were observed after a reduction in the link stability value to an average of 89%. Thus, this value of 0.89 will be the threshold of link stability in control program: variable thresholdStab in Algorithm 1.

Unlike the previous results and following the architecture of Figure 1, in this final test, the controller selected the PV value based on the stability of the links. The ESP32 controller received the RSSI values of the links, stored the values and calculated the stability values of these links in real time to detect whether a variable change was required (PV1 or PV2).

**Figure 14:**
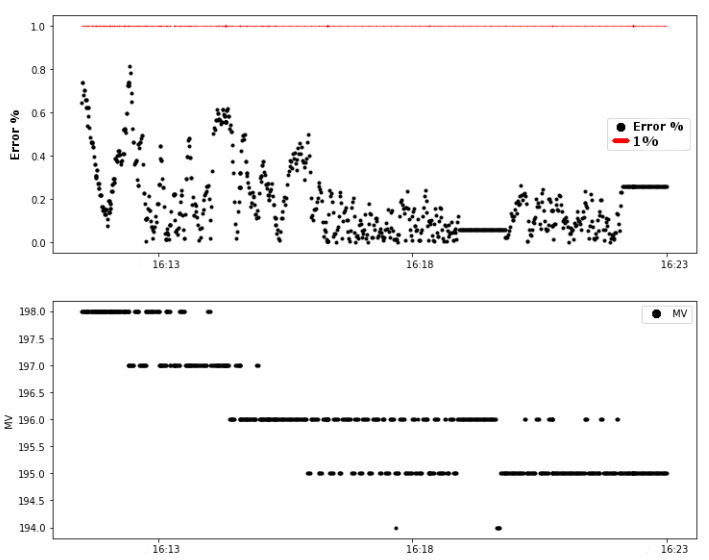
Result of the control system based on link stability: error (%) and MV.

Figure 14 shows that the error (%) of the controller decreased after a period of time until the end of the test.

The result shows that the permanence of the error below the maximum value, with low variation, ensures that the manipulated variable of the control loop remains regulated.

In this last test, the same attenuation procedures were performed for the link of the level measurement sensor. The error remained below the maximum limit due to the implementation of control logic based on link stability.

**Figure 15:**
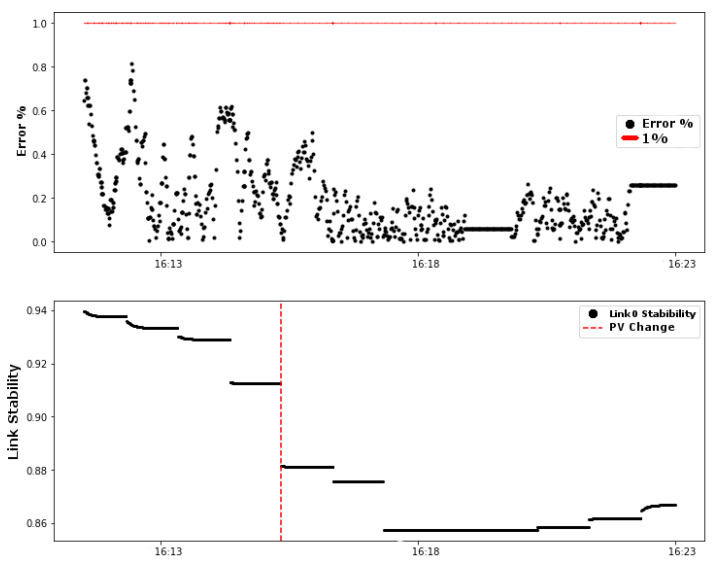
Results of the control system based on link stability: error (%) and link stability.

By determining the link stability values equal to or less than 89%, the controller changes the choice of the PV value. This behavior can be observed in Figure 15.

The dashed vertical red line in Figure 15 indicates the time the controller detected the link stability value less than 89% and changed the PV value to the value of the second link (Link2). This change kept the error below the threshold.

Finally, the ISA 100.11a network control system was implemented and the link stability metric was able to identify possible instabilities and prevent the failure of the system’s control loop.

There are few works that performed experiments with networked control systems using WirelessHART and ISA 100.11a protocols. In addition, there is no work that implemented a WNCS based on the link stability parameter. Thus, a comparison between researches was not possible.

## 6. Conclusions

In this paper, we present the implementation of an ISA 100.11a networked control system. ISA 100.11a networks are widely used in monitoring applications. However, control applications require careful attention when designing communication and control systems. Thus, the first contribution of this work was the evaluation of networked control systems using the ISA 100.11a protocol.

The system controller uses link stability as a decisive factor in choosing PV values. The link stability model is able to detect instabilities in the communication between the instruments, and consequently, to predict failures in the control loop. Some preliminary tests were performed to analyze the behavior of the control system from the generation of purposeful noise in the system. Purposeful noises reduced the value of the link stability and then increased the error of the control loop. Thus, the proposed WCNS is based on the link stability to avoid failure in the control system. When the controller took into account the link stability, the system tests showed satisfactory results. The controller detected a low stability of the sensor link and changed the PV value to another link. The detection of link instability kept the control loop within the desirable limits. The second contribution was the use of the link stability model in a wireless network control system.

The tests were performed with ISA 100.11a instruments from the manufacturer Yokogawa Eletric. Additionally, the monitoring of the network and control system variables was done from the interface with Modbus TCP and GSAP commands. Thus, the third main contribution of this work was to provide experimental tests with instruments from manufacturers in the market.

## Figures and Tables

**Table 1:** Papers that define link stability and its parameters.

Paper	Year	Distance between Nodes	Link Expiration Time	Packet Delivery Rate	Received Signal Strength	Sensor Networks
Zou and Tao [18]	2012	✓	N	N	N	N
Sun et al. [19]	2010	✓	N	N	N	N
Boukerche et al. [20]	2017	✓	N	N	N	N
De Rango and Francesca [21]	2012	✓	N	N	N	N
Zhang et al. [22]	2012	✓	N	N	✓	N
Sharma and Pathak [23]	2015	N	✓	N	N	N
Idoudi et al. [24]	2016	N	✓	N	N	N
Chama et al. [25]	2013	N	✓	N	N	N
Al-Qhdah et al. [26]	2016	N	✓	N	N	N
Wang et al. [27]	2010	N	✓	N	N	N
Li and Yan [28]	2017	N	N	✓	✓	N
Priya et al. [29]	2017	N	N	✓	N	✓
Wenqing [30]	2011	N	N	N	✓	N
Brahmbhatt et al. [16]	2015	N	N	N	✓	N
Patil and Patil [31]	2015	N	N	N	✓	N
**Model proposed: Florencio and Neto [17]**	**2019**	N	N	✓	✓	✓
✓: Covered				N: Not Covered		

**Table 2:** ISA 100.11a gateway Modbus memory mapping.

TAG	Modbus Data Type	Offset	Description
PV1	Holding registers	13	LD01 Sensor Process Variable
	Holding registers	14	
PV2	Holding registers	34	LD02 Sensor Process Variable
	Holding registers	35	

**Table 3:** Controller Modbus memory mapping.

TAG	Data Type Modbus	Description
ModePID	Holding registers	Mode to enable setpoint change
PV1	Holding registers	LD01 sensor process variable
SP	Holding registers	Setpoint
MV	Holding registers	Manipulated variable (PID output)
PV2	Holding registers	LD02 sensor process variable
$RSSI_{\alpha }$	Holding registers	RSSI do link α
$DPDUTx_{\alpha }$	Holding registers	Number of packages delivered by the link α
$DPDUTxFail_{\alpha }$	Holding registers	Number of packages lost by the link α

**Table 4:** GSAP services.

Service	Command
*Session*	*G_Session request*
	*G_Session confirm*
*Topology_Report*	*G_Topology_Report request*
	*G_Topology_Report confirm*
*Device_List_Report*	*G_Device_List_Report request*
	*G_Device_List_Report confirm*
*Device_Health_Report*	*G_Device_Health_Report request*
	*G_Device_Health_Report confirm*
*Neighbor_Health_Report*	*G_Neighbor_Health_Report request*
	*G_Neighbor_Health_Report confirm*
*Network_Health_Report*	*G_Network_Health_Report request*
	*G_Network_Health_Report confirm*

**Table 5:** GSAP command fields: *Neighbor_Health request*.

Field	Size (Bytes)	Description	Example Value
Version	1	Gateway version	OxFO
Service type	1	Command code	0x07
Session ID	4	Session identifier. Value generated after the session creation	–
Transaction ID	4	Each communication in a session generates a different ID	–
Data size	4	Size of the object to request data	–
Header CRC	4	Header check code. Header: version, type, session ID, transaction ID and data size	–
Network address	16	Network address of the instrument that you want to collect information from neighbors	–
Data CRC	4	Network address field check code	–

**Table 6:** Preliminary test results.

Test	Time to Instability	Reduction Value Range (Link Stability)
01	5m	90% a 86% (Median: 88%)
02	3m	92% a 88% (Median: 90%)
03	5m	92% a 85% (Median: 89%)
04	7m	92% a 87% (Median: 89%)
05	7m	88% a 86% (Median: 87%)
06	4m	89% a 86% (Median: 88%)
07	3m	92% a 90% (Median: 91%)
